# Decreasing skeletal muscle as a risk factor for mortality in elderly patients with sepsis: a retrospective cohort study

**DOI:** 10.1186/s40560-016-0205-9

**Published:** 2017-01-11

**Authors:** Keita Shibahashi, Kazuhiro Sugiyama, Masahiro Kashiura, Yuichi Hamabe

**Affiliations:** Department of Emergency and Intensive Care Center, Tokyo Metropolitan Bokutoh Hospital, 4-23-15, Kotobashi, Sumida-ku, Tokyo, 130-8575 Japan

**Keywords:** Sepsis, Sarcopenia, Intensive care, Mortality

## Abstract

**Background:**

Older patients account for the majority of patients with sepsis. The objective of this study was to determine if decreased skeletal muscle mass is associated with outcomes in elderly patients with sepsis.

**Methods:**

Patients (60 years and older) who were admitted to a tertiary medical center intensive care unit with a primary diagnosis of sepsis between January 2012 and February 2016 were included. Patients who had not undergone abdominal computed tomography on the day of admission, had cardiopulmonary arrest on arrival, or had iliopsoas abscess were excluded from the analyses. Cross-sectional muscle area at the 3rd lumber vertebra was quantified, and the relation to in-hospital mortality was analyzed. Multivariable logistic regression analysis that included sex and APACHE II score as explanatory variables was performed. The optimal cutoff value to define decreased muscle mass (sarcopenia) was calculated using receiver operating characteristic curve analysis, and the odds ratio for in-hospital mortality was determined.

**Results:**

There were 150 elderly patients with sepsis (median age, 75 years) enrolled; in-hospital mortality and median APACHE II score were 38.7 and 24%, respectively. The skeletal muscle area of deceased patients was significantly lower than that of the survival group (*P* < 0.001). The multivariable logistic regression analysis demonstrated that decreased muscle mass was significantly associated with increased mortality (odds ratio = 0.94, 95% confidence interval = 0.90 to 0.97, *P* < 0.001). The optimal cutoff value of skeletal muscle area to predict in-hospital mortality was 45.2 cm^2^ for men and 39.0 cm^2^ for women. With these cutoff values, the adjusted odds ratio for decreased muscle area was 3.27 (95% CI, 1.61 to 6.63, *P* = 0.001).

**Conclusions:**

Less skeletal muscle mass is associated with higher in-hospital mortality in elderly patients with sepsis. The results of this study suggest that identifying patients with low muscularity contributes to better stratification in this population.

## Background

Sepsis is a clinical syndrome characterized by physiologic, biologic, and biochemical abnormalities caused by a dysregulated inflammatory response to infection. It also includes organ dysfunction attributed to it and is a major public health concern. The reported incidence is disproportionately higher in older patients. Older patients account for the majority (60–85%) of all episodes, and this figure is likely to increase in the future [1–3]. Therefore, the importance of accurate stratification of elderly patients with sepsis is growing, as it can aid in developing a treatment strategy, allocating healthcare resources, and assessing the effectiveness of novel therapies. While chronologic age is an important element in assessing the anomaly in the host’s inflammatory response, physiologic age is a more important determinant of outcomes. Recently, sarcopenia, which is defined as the loss of skeletal muscle mass and strength with advancing age [4], has increasingly been recognized as an important factor that can act as a marker of decreased physiologic reserve because it is highly important for immune function, glucose disposal, protein synthesis, and mobility [5, 6].

We hypothesized that decreased skeletal muscle mass is a predictive marker for the outcome of elderly patients with sepsis. In this retrospective study, we investigated whether decreased skeletal muscle mass is associated with in-hospital mortality in elderly patients with sepsis.

## Methods

### Population cohort and data acquisition

Patients who were admitted to a tertiary medical center intensive care unit (ICU) with a primary diagnosis of sepsis between January 2012 and February 2016 were retrospectively identified. Our tertiary medical center admits only severe patients either directly transferred from the scene by an emergency response team or referred from another medical facility. We employ a strategy, wherein, we aggressively perform torso CT on almost all patients. This entails confirmation of the potential source of infection, assessment of anatomical structures of the lesion in detail, and identification of any coexisting lesions as reliably and immediately as possible. Elderly (60 years and older) patients were included in the analysis. Patients were excluded from the analysis who had not undergone abdominal computed tomography (CT) on the day of admission or had cardiopulmonary arrest on arrival (CPAOA). Because updated definitions of sepsis that offer greater consistency for clinical trials were released in 2016 [7], we determined whether the patients met the revised criteria for sepsis; otherwise, they were excluded from the study. Patients with iliopsoas abscess were also excluded from the analysis because of the possible confounding effect on skeletal muscle area.

Demographic and clinical data included age, sex, body weight, Glasgow coma scale (GCS) score, body temperature, mean arterial pressure, heart rate, respiratory rate, arterial oxygen partial pressure, fractional inspired oxygen, blood test results (platelets, bilirubin, creatinine, Na, K, hematocrit, white blood cell count, and lactate on admission), administration of vasopressors, length of stay (LOS) in the hospital, LOS in the ICU, in-hospital mortality, location of death, infection source, chronic health problems, and recent surgery. We calculated the Sepsis-related Organ Failure Assessment (SOFA) score [8], which determines the extent of a patient’s organ failure based on the respiratory, cardiovascular, hepatic, coagulation, renal, and neurological systems, as well as the second version of the Acute Physiology and Chronic Health Evaluation (APACHE II) score [9], which determines the severity of disease based on a patient’s age and physiological measurements.

### Computed tomography measurement

**Fig. 1:**
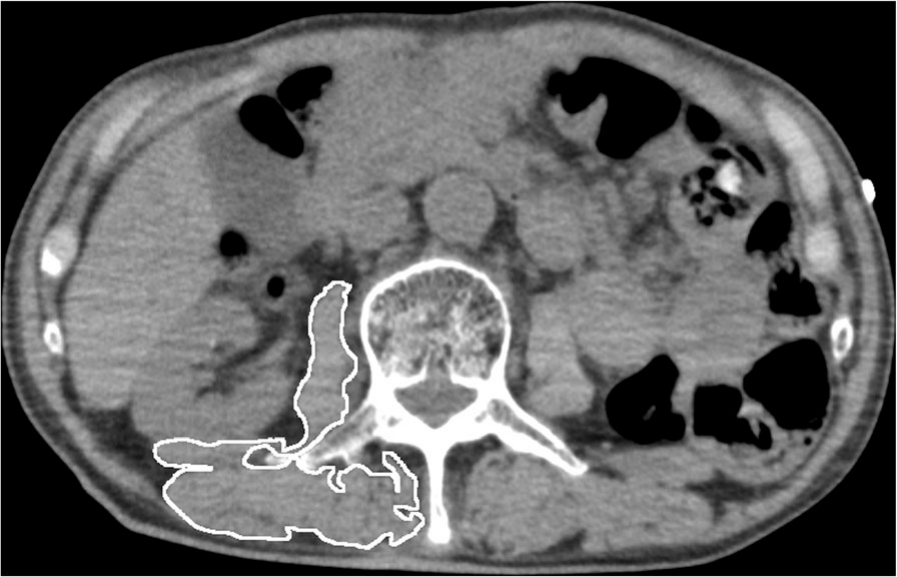
CT image at the L3-sectional muscle. The right L3-sectional muscle area is outlined. Muscle area and mean muscle attenuation are calculated by the picture archiving and communication system (PACS) software

Access to computed tomography (CT) images on the day of admission was available through the picture archiving and communication system (PACS; SYNAPSE software, Fujifilm Medical Co., Tokyo, Japan). After the observer outlined the muscle, the range of the area of interest was calculated by the software. Muscle attenuation, a measure of muscle density and fatty infiltration, was quantified using Hounsfield units (HU) obtained from the CT image. Lean skeletal muscle mass was estimated by measuring the cross-sectional area of the psoas and paraspinal (quadratus lumborum, erector spinae) muscles at the third lumber vertebra (L3) as shown in Fig. 1. A strong correlation between the cross-sectional area of skeletal muscle at this landmark and whole-body muscle distribution has been reported, and the validity and reliability of this method to estimate lean skeletal muscle mass have been established [10–12]. HUs within the range of interest of each muscle were calculated, and the average value was used for the final muscle attenuation. The observer was blinded to patients’ survival status.

### Statistical analysis

Patients were grouped according to outcome (survival or deceased). For descriptive statistics, numeric or ordered variables are presented as medians with interquartile ranges and were compared using Mann–Whitney *U* tests. Categorical variables are presented as counts and percentages and were tested for significance using Fisher’s exact tests. We used a multivariable logistic regression model to determine whether decreased skeletal muscle area is independently associated with in-hospital mortality in patients with sepsis. We also performed additional stratified analyses that divide patients into two groups of 60–80 years and over 80 years to adjust heterogeneity because muscle mass of over 80 years is lost at an accelerated rate (15%/year) compared to the rate of approximately 8% per decade from the age of 50 years [13]. To minimize the risk of falsely identifying significant results, explanatory variables were predetermined before the analysis based on previous studies [9, 14–16]. In addition to the skeletal muscle area, the APACHE II score and sex were included in the model. To test multicollinearity, we evaluated the variance inflation factor. To determine the optimal cutoff value of skeletal muscle area for predicting in-hospital mortality and the area under the curve (AUC), we performed receiver operating characteristic (ROC) curve analysis. We defined the patients with a skeletal muscle area lower than the cutoff value as sarcopenic, and the odds ratio for mortality was determined. For each groups, event-time distributions were estimated with the use of the Kaplan–Meier method. The difference of estimated survival rate was tested using log-rank test. We performed an analysis using Cox proportional hazards regression and taking time to in-hospital death as the dependent variable and sarcopenia as the main predictor variable. To adjust for potential confounders, APACHE II score and sex were included in a multivariable model. Hazard ratios and 95% confidence interval (CI) are given. All statistical tests were two-tailed, and *P* values <0.05 were considered significant.

All statistical analyses were performed with EZR (Saitama Medical Center, Jichi Medical University, Saitama, Japan), which is a graphical user interface for R (The R Foundation for Statistical Computing, Vienna, Austria). More precisely, it is a modified version of R commander designed to add statistical functions frequently used in biostatistics [17].

## Results

During the study period, 168 patients met the inclusion criteria. Of those, patients were excluded for the following reasons: *n* = 4, no abdominal CT; *n* = 10, CPAOA; and *n* = 1, had an iliopsoas abscess. Of the remaining 153 patients, 3 patients who did not meet the recent criteria for sepsis were excluded, resulting in 150 patients (103 men, 47 women; age 75 [68, 82] years) in the analysis. All the predictors required to calculate the APACHE II and SOFA scores were present in the dataset.

The clinical characteristics and radiological features are summarized in Table 1. The overall APACHE II score was 24 [18, 19], and the in-hospital mortality rate was 38.7%. Lactate level on admission was 4.2 [2.3, 7.2], and that of 116 patients (77%) were higher than 2.0 mmol/L. The survival group included 92 patients, and the deceased group included 58 patients. Of the 58 deceased patients, 44 deceased at ICU and 14 deceased after transfer to general floor. The APACHE II score was significantly higher in the deceased group (*P* = 0.002).

**Table 1:** Patient characteristics and radiological findings

	All patients	Group		*P* value
		Survival	Deceased	
Number of patients	150	92	58	
Location of death				
ICU			44	
After transferred to general floor			14	
Age (years)	75 [68, 82]	74 [67, 81]	77 [71, 83]	0.13
Sex, men (%)	103 (69)	63 (69)	40 (69)	0.99
GCS	14 [9, 15]	14 [10, 15]	12 [7, 15]	0.21
Lactate on admission (mmol/L)	4.2 [2.3, 7.2]	3.9 [1.8, 6.3]	5.9 [2.4, 8.9]	0.053
Infection source (%)				0.33
Lung	45 (30)	23 (25)	22 (38)	
Urinary tract	32 (21)	24 (26)	8 (14)	
Peritonitis/abscess	16 (11)	8 (9)	8 (14)	
Perforated viscus	15 (10)	10 (11)	5 (9)	
Cholecystitis/cholangitis	10 (7)	8 (9)	2 (3)	
Unknown	9 (6)	4 (4)	5 (9)	
NecFas/decubitus ulcer	8 (5)	5 (5)	3 (5)	
Ischemic bowel	5 (3)	3 (3)	2 (3)	
Colitis	5 (3)	4 (4)	1 (2)	
Blood stream infection	3 (1)	1 (1)	2 (3)	
Bone/joint	1 (1)	1 (1)	0 (0)	
CNS	1 (1)	1 (1)	0 (0)	
SOFA score	9 [7, 12]	8 [7, 11]	10 [8, 12]	0.004
APACHE II score	24 [19, 30]	23 [18, 27]	26 [22, 34]	0.002
Body weight (kg)	54 [48, 60]	54 [48, 61]	54 [48, 58]	0.42
Skeletal muscle area (cm^2^)	41.6 [34.0, 48.1]	43.3 [36.5, 50.9]	36.8 [30.1, 43.2]	<0.001
Muscle attenuation (HU)	32.6 [24.1, 39.9]	33.2 [24.2, 40.0]	31.0 [23.6, 39.8]	0.38
LOS in ICU	8 [4, 15]	9 [6, 15]	5 [2, 14]	
LOS in hospital	27 [7, 51]	40 [18, 62]	8 [3, 25]	

*ICU* intensive care unit, *GCS* Glasgow coma scale, *NecFas* necrotizing fasciitis, *CNS* central nervous system, *SOFA* Sepsis-related Organ Failure Assessment, *APACHE II* second version of the acute physiology and chronic health evaluation, *HU* Hounsfield unit, *LOS* length of stay

**Fig. 2:**
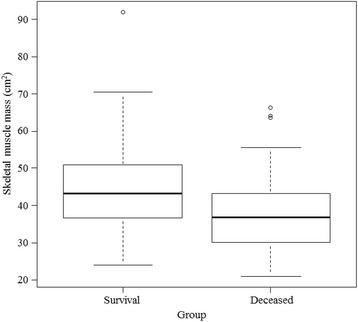
Comparison of the skeletal muscle area between survival group and deceased group. Skeletal muscle area was significantly larger in survival group (43.3 [36.5, 50.9] vs 36.8 [30.1, 43.2], *P* < 0.001)

The median skeletal muscle area in all patients was 41.6 [34.0, 48.1] cm^2^. Those of the survival group was significantly larger than the deceased group (43.3 [36.5, 50.9] and 36.8 [30.1, 43.2] cm^2^, *P* < 0.001; Fig. 2). The SOFA score of the deceased group was significantly higher than that of the survival group (*P* = 0.004).

The results of the multivariable regression analysis are shown in Table 2. The variance inflation factors for multicollinearity were lower than 1.06 among the predetermined explanatory variables. Among the variables, skeletal muscle area (cm^2^) was an independent predictive parameter for in-hospital mortality, and the adjusted odds ratio was 0.94 (95% CI, 0.90 to 0.97, *P* < 0.001). In the stratified analysis, the association between skeletal muscle area and in-hospital mortality was significant in both groups of 60–80 years and over 80 years, and the adjusted odds ratio was 0.96 (95% CI, 0.92 to 0.99, *P* = 0.032) and 0.89 (95% CI, 0.81 to 0.98, *P* = 0.016), respectively.

**Table 2:** Results of multivariable logistic regression analysis to determine variables independently associated with in-hospital mortality of patients with sepsis

	Odds ratio (95% confidence interval)	*P* value
Overall analysis		
Skeletal muscle area (cm^2^)	0.94 (0.90–0.97)	<0.001
APACHE II score	1.07 (1.02–1.12)	0.003
Sex, men	1.46 (0.67–3.22)	0.34
Stratified analysis		
60–80 years		
Skeletal muscle area (cm^2^)	0.96 (0.92–0.99)	0.032
Over 80 years		
Skeletal muscle area (cm^2^)	0.89 (0.81–0.98)	0.016

*APACHE II* second version of the Acute Physiology and Chronic Health Evaluation

**Fig. 3:**
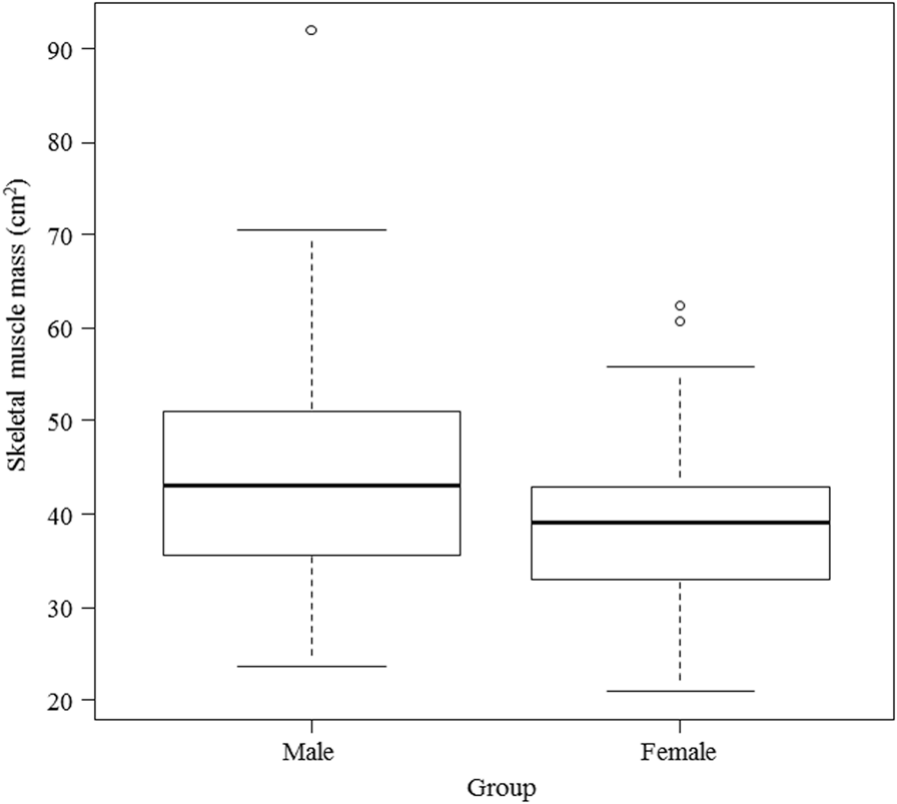
Comparison of the skeletal muscle area between men and women. Skeletal muscle area was significantly larger in men (43.1 [35.5, 51.0] vs 39.0 [33.1, 43.0], *P* = 0.005)

**Fig. 4:**
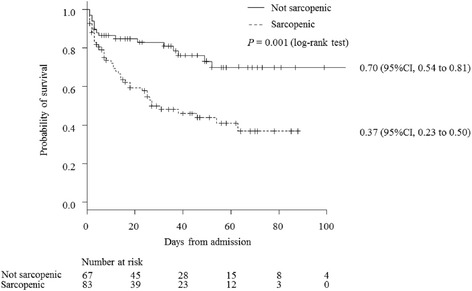
Kaplan–Meier estimates of time to death. Estimated survival rate at 100 days after admission was significantly different between two groups (log-rank test *P* = 0.001). Risk of in-hospital mortality was significantly higher for sarcopenic patients even when adjusted for APACHE II and sex (Cox proportional hazards regression analysis, hazard ratio 2.55, *P* = 0.001)

The cutoff value of skeletal muscle area to predict in-hospital mortality was calculated for each sex because the skeletal muscle area was significantly larger in men than in women (43.1 [35.5, 51.0] vs 39.0 [33.1, 43.0] cm^2^, *P* = 0.005; Fig. 3). ROC curve analysis demonstrated that the optimal cutoff values were 45.2 cm^2^ for men and 39.0 cm^2^ for women, and the AUCs were 0.65 (95% CI, 0.54 to 0.76) and 0.72 (95% CI, 0.58 to 0.88), respectively. Based on our definition of sarcopenia (using the cutoff value), in-hospital mortality was significantly higher for patients with sarcopenia, and the adjusted odds ratio was 3.27 (95% CI, 1.61 to 6.63, *P* = 0.001). Kaplan–Meier estimates of mortality were drown for each group (Fig. 4). The estimated survival rate 100 days after admission was 0.70 (95% CI, 0.54 to 0.81) for not sarcopenic patients and 0.37 (95% CI, 0.23 to 0.50) for sarcopenic patients, and the difference was significant (*P* < 0.001). Cox proportional hazards regression analysis showed a higher risk of in-hospital mortality for sarcopenic patients even when adjusted for APACHE II score and sex, and the hazard ratio was 2.55 (95% CI, 1.43 to 4.56, *P* = 0.001).

## Discussion

Sarcopenia is important as an independent predictor of falls, disability, loss of independence, and increased mortality. While the prognostic value of sarcopenia has been determined for patients after surgery, trauma, or with cancer [9–12, 18, 20–25], its importance for patients with sepsis has not been evaluated. In the present sample of elderly patients with sepsis admitted to a tertiary medical center ICU, decreased skeletal muscle mass was a significant predictor of in-hospital mortality. This is the first study, to the best of our knowledge, to examine the implications of sarcopenia in elderly patients with sepsis.

A wide range of techniques can be used to assess muscle mass. CT and magnetic resonance imaging (MRI) are considered to be very precise imaging systems and as gold standards for estimating muscle mass. In this study, we used the cross-sectional area of the muscle determined using CT; this provides an estimation of the overall muscle mass and has been used in a variety of studies to predict lean muscle mass [10–12]. While it is difficult to perform these measurements for the sole purpose of estimating skeletal muscle mass, CT is frequently required in patients with sepsis as a part of the initial work up; therefore, an early assessment of muscularity in this patient population is possible, and the cross-sectional view of the muscle provides an easily obtained objective method for estimating lean muscle mass in these patients. Because it takes only a few minutes, it can be easily performed in most clinical scenarios. A strength of this study is the ease of incorporating our findings into practice.

Clinical characteristics that impact the severity of sepsis and outcome include the host’s response to infection, site and type of infection, and therapeutic strategy. The therapeutic strategy for sepsis has been standardized in practice guidelines; several studies have reported decreasing sepsis-related mortality rates over time with the implementation of therapeutic strategies, after adjusting for multiple variables, suggesting improvement owing not only to sepsis criteria and to the progress in medicine in general but also to these strategies [26, 27]. Furthermore, the importance of the host response as a prognostic factor for patients with sepsis is growing. Among the factors related with host response, age is reportedly a primary risk factor for mortality because of its association with comorbid illnesses, impaired immunologic responses, malnutrition, and increased exposure to potentially resistant pathogens. Although chronologic age is a good objective marker of the anomaly in the host response and included in scoring models of prognosis for patients in the ICU, considerable individual variation in physical condition exists among the elderly. While muscle mass is generally lost with aging and the prevalence of sarcopenia ranges between 5 and 13% in older people, the severity of muscle loss significantly varies in this population. In the current study, we confirmed associations between decreasing muscle mass and sepsis related mortality. Because skeletal muscle atrophy can cause physical decline such as impaired cytokine [28] and insulin signaling [19, 29] that may result in glucose intolerance, we speculate that stratification by muscle mass may reflect physical age and help circumvent the difficulties associated with prognostication and classification of elderly patients with sepsis by chronologic age. The mechanisms for the relationship between sarcopenia and poor prognosis cannot be definitively determined because of the study design. However, the results of the current study highlight the need to prevent progressive loss of skeletal muscle mass and function in the elderly.

The literature suggests that the combination of exercise and nutrition is the key intervention for preventing, treating, and slowing down the progress of sarcopenia [30, 31]. Resistance exercise combined with protein supplementation at higher protein doses of 40 g leads to greater muscle gain than exercise or protein supplementation alone in the elderly [31, 32]. The awareness of the benefits of exercise and diet is not enough in older people. Enhancing participation in exercise for older patients might prevent sarcopenia and improve sepsis outcomes in the elderly population. Rehabilitation and nutritional strategies that focus on preventing muscle loss may also contribute to better outcomes. Further research is required to test these hypotheses; however, the results of the present study highlight the importance of muscle mass in patients with sepsis.

### Limitations

Owing to the retrospective design, biases and confounding are major concern. We tried to adjust for possible confounders such as age, comorbidity, recent surgery, and sex by performing multivariable regression analysis using the APACHE II score, which is calculated with these variables, and sex as explanatory variables. This study was performed at a single center with a small sample. Because it is our strategy to aggressively perform torso CT for patients with sepsis, almost all patients including those with lung infection, CNS infection, and necrotizing fasciitis/decubitus ulcer undergo abdominal CT at our ICU. Some institutes may not perform abdominal CT for such cases. Hence, the infection source of patients with abdominal CT may differ between other studies and the present study, limiting external validity of our results. We expect similar associations between muscle mass and in-hospital mortality in other cohorts; however, the optimal cutoff value should be validated. We report that the odds ratio for in-hospital mortality was 3.27, but the 95% CI was wide, with a minimum value of 1.61. Further research with a larger sample is necessary to establish the clinical significance of sarcopenia.

## Conclusions

We found that less skeletal muscle mass is associated with higher in-hospital mortality of elderly patients with sepsis. The results of this study suggest that identifying patients with low muscularity may contribute to better stratification of elderly patients.

## Abbreviations

APACHE II: Second version of the Acute Physiology and Chronic Health Evaluation
AUC: Area under the curve
CPAOA: Cardiopulmonary arrest on arrival
CT: Computed tomography
GCS: Glasgow coma scale
HU: Hounsfield unit
ICU: Intensive care unit
LOS: Length of stay
MRI: Magnetic resonance imaging
ROC: Receiver operating characteristic
SOFA: Sepsis-related Organ Failure Assessment
